# 85 Sole Propofol Use in Burn Patients Is Associated with Better Postprocedural Outcomes Than Using Propofol-Ketamine

**DOI:** 10.1093/jbcr/irae036.084

**Published:** 2024-04-17

**Authors:** Yash Ramgopal, Christopher G Richter, Sunskruthi Krishna, Suhaib Shah, Carolina Segura, Isabel B Obias, Dalton Amador, Jean pierre Durand, Amina E I ayadi, Georgiy Golovko, Juquan Song, Steven E Wolf

**Affiliations:** University of Texas Medical Branch, Houston, TX; University of Texas Medical Branch, Galveston, TX; University of Texas Medical Branch at Galveston, Galveston, TX; University of Texas Medical Branch, Blocker Burn Unit, Galveston, TX; University of Texas Medical Branch, Houston, TX; University of Texas Medical Branch, Galveston, TX; University of Texas Medical Branch at Galveston, Galveston, TX; University of Texas Medical Branch, Blocker Burn Unit, Galveston, TX; University of Texas Medical Branch, Houston, TX; University of Texas Medical Branch, Galveston, TX; University of Texas Medical Branch at Galveston, Galveston, TX; University of Texas Medical Branch, Blocker Burn Unit, Galveston, TX; University of Texas Medical Branch, Houston, TX; University of Texas Medical Branch, Galveston, TX; University of Texas Medical Branch at Galveston, Galveston, TX; University of Texas Medical Branch, Blocker Burn Unit, Galveston, TX; University of Texas Medical Branch, Houston, TX; University of Texas Medical Branch, Galveston, TX; University of Texas Medical Branch at Galveston, Galveston, TX; University of Texas Medical Branch, Blocker Burn Unit, Galveston, TX; University of Texas Medical Branch, Houston, TX; University of Texas Medical Branch, Galveston, TX; University of Texas Medical Branch at Galveston, Galveston, TX; University of Texas Medical Branch, Blocker Burn Unit, Galveston, TX; University of Texas Medical Branch, Houston, TX; University of Texas Medical Branch, Galveston, TX; University of Texas Medical Branch at Galveston, Galveston, TX; University of Texas Medical Branch, Blocker Burn Unit, Galveston, TX; University of Texas Medical Branch, Houston, TX; University of Texas Medical Branch, Galveston, TX; University of Texas Medical Branch at Galveston, Galveston, TX; University of Texas Medical Branch, Blocker Burn Unit, Galveston, TX; University of Texas Medical Branch, Houston, TX; University of Texas Medical Branch, Galveston, TX; University of Texas Medical Branch at Galveston, Galveston, TX; University of Texas Medical Branch, Blocker Burn Unit, Galveston, TX; University of Texas Medical Branch, Houston, TX; University of Texas Medical Branch, Galveston, TX; University of Texas Medical Branch at Galveston, Galveston, TX; University of Texas Medical Branch, Blocker Burn Unit, Galveston, TX; University of Texas Medical Branch, Houston, TX; University of Texas Medical Branch, Galveston, TX; University of Texas Medical Branch at Galveston, Galveston, TX; University of Texas Medical Branch, Blocker Burn Unit, Galveston, TX; University of Texas Medical Branch, Houston, TX; University of Texas Medical Branch, Galveston, TX; University of Texas Medical Branch at Galveston, Galveston, TX; University of Texas Medical Branch, Blocker Burn Unit, Galveston, TX

## Abstract

**Introduction:**

Propofol and its combination with ketamine have been used as sedatives in burn patients undergoing operative procedures such as surgical debridement and wound care. Although research has been conducted on comparing the complications of propofol-ketamine and sole propofol use in the emergency department (ED), limited research is available on the use of these sedatives specifically in burn patients who are in a hypermetabolic state. Our study aims to bridge this gap by comparing the postprocedural outcomes of using propofol-ketamine and propofol alone during operative procedures in pediatric and adult burn patients.

**Methods:**

Data for this study was obtained from a national database of de-identified medical records. Two burn patient populations undergoing operative procedures were identified based on sedative use: propofol-ketamine and propofol alone. Patients with a past medical history of respiratory and cardiovascular conditions were excluded. Cohorts were balanced using propensity score matching of covariates: age at index, sex, race, and ethnicity. Five complications were studied 0-14 days post-administration of sedative: anxiety, hyperkalemia, renal failure, hypotension, and respiratory failure. Statistical analysis included risk differences with a significance value of p < 0.05.

**Results:**

We identified 7,993 patients who were administered only propofol during operative procedures and 7,148 patients who were administered the combination of propofol and ketamine. Propofol-ketamine use had a statistically significant higher incidence of anxiety (2.91% vs 1.62%, p < 0.0001), hyperkalemia (1.19% vs 0.81%, p = 0.0402), renal failure (2.58% vs 1.83%, p = 0.0037), hypotension (1.56% vs 1.10%, p = 0.0247), and respiratory failure (3.40% vs 2.80%, p = 0.0488).

**Conclusions:**

Our study indicates that sole propofol use in burn patients undergoing operative procedures is associated with better postprocedural outcomes than using a combination of propofol and ketamine. Indicators of propofol infusion syndrome (hyperkalemia and renal failure), which is a life-threatening rare complication of the sedative, are higher with the use of propofol-ketamine. Future directions of the study include analyzing the relationship of burn severity (measured by % total burn surface area) on outcomes in the two cohorts.

**Applicability of Research to Practice:**

Wound dressing changes and surgical excision in burn patients often provoke considerable pain and anxiety, hence sedatives such as propofol and ketamine are routinely used to ease their concerns. The findings from this study have the potential to help physicians choose the right sedative by balancing patient comfort and the risk of adverse effects.

